# Correction: Sex Ratio and Body Mass of Adult Herbivorous Beetles Depend on Time of Occurrence and Light Conditions

**DOI:** 10.1371/journal.pone.0147891

**Published:** 2016-01-22

**Authors:** Adrian Łukowski, Ewa Mąderek, Marian J. Giertych, Piotr Karolewski

## Notice of Republication

This article was republished on January 7, 2016, to correct spacing errors in the text that were introduced during the pre-production process. Please download this article again to view the correct version. The originally published, uncorrected article and the republished, corrected article are provided here for reference.

## Supporting Information

S1 File Originally published, uncorrected article. (PDF)

S2 File Republished, corrected article. (PDF)

## References

[1] Łukowski A, Mąderek E, Giertych MJ, Karolewski P (2015) Sex Ratio and Body Mass of Adult Herbivorous Beetles Depend on Time of Occurrence and Light Conditions. PLoS ONE 10(12): e0144718 doi: 10.1371/journal.pone.0144718 26657564 10.1371/journal.pone.0144718 PMC4682288

