# Supplementary material for: Correction: Sex Ratio and Body Mass of Adult Herbivorous Beetles Depend on Time of Occurrence and Light Conditions
Source: PLoS One. 2016 Jan 22;11(1):e0147891. doi: 10.1371/journal.pone.0147891 (PMC4723360; doi:10.1371/journal.pone.0147891)
Supplement: S1 File — (PDF) [file pone.0147891.s001.pdf]

PLOS ONE

RESEARCH ARTICLE

Sex Ratio and Body Mass of Adult Herbivorous Beetles Depend on Time of Occurrence and Light Conditions

Adrian Łukowski[1,2]*, Ewa Mąderek[1], Marian J. Giertych[1,3], Piotr Karolewski[1]

1 Institute of Dendrology, Polish Academy of Sciences, Kórnik, Poland, **2** Department of Forest Protection, Faculty of Forestry, Poznań University of Life Sciences, Poznań, Poland, **3** Faculty of Biological Sciences, University of ZielonaGóra, ZielonaGóra, Poland

* adrian.lukowski@gmail.com

CrossMark
click for updates

OPEN ACCESS

Citation: Łukowski A, Mąderek E, Giertych MJ, Karolewski P (2015) Sex Ratio and Body Mass of Adult Herbivorous Beetles Depend on Time of Occurrence and Light Conditions. PLoS ONE 10(12): e0144718. doi:10.1371/journal.pone.0144718

Editor: Kyung-Jin Min, Inha University, REPUBLIC OF KOREA

Received: August 28, 2015

Accepted: November 23, 2015

Published: December 14, 2015

Copyright: © 2015 Łukowski et al. This is an open access article distributed under the terms of the Creative Commons Attribution License, which permits unrestricted use, distribution, and reproduction in any medium, provided the original author and source are credited.

Data Availability Statement: All relevant data are within the paper.

Funding: The research was financially supported by the National Science Centre, Poland (grant no. N N304 037640 for PK), the statutory activities of the Institute of Dendrology Polish Academy of Sciences in Kórnik, and grant for young scientists (for AŁ; Poznan University of Life Sciences, Poland). The funders had no role in study design, data collection and analysis, decision to publish, or preparation of the manuscript.

Abstract

Body mass and sex ratio (F/M) of folivorous insects are easily measured parameters that are commonly used to assess the effect of food quality, living conditions, and preferences on the selection of favourable sites for offspring. A study was conducted on the polyphagous beetle, *Gonioctenaquinquepunctata* (a pest of the native *Prunus padus* and alien *P. serotina*) and on the monophagous beetle, *Alticabrevicollis coryletorum* (a pest of *Corylus avellana*). Both species have a similar life cycle with emergence of current-year adults in summer, and reproduction of 1-year-old insects in spring. *A. brevicollis coryletorum* feeds primarily on sunlit shrubs, while *G. quinquepunctata* prefers shaded leaves. The present study assessed the effect of time of occurrence(insect age) on body mass in both sexes and on the sex ratio F/M, taking into account the influence of light conditions associated with their favoured food source (sunlit vs. shaded leaves). We hypothesized that a change in body mass in current-year insects would be determined by the amount of consumed food, while the sex ratio would be stable, when in 1-year-old insects females would die shortly after oviposition, while males would be active for a prolonged time. Results confirmed the hypothesis that changes in mass of current-year beetles was determined by the amount of food intake. We also found that in spring, unfertilized females coexist with fertilized ones and that the latter females live for some time after oviposition; resulting in fluctuations of the mean mass for females. In both species, 1-year-old beetles were heavier than current-year. The preference of *A. brevicollis coryletorum* for sunlit leaves results in a higher body weight than in *G. quinquepunctata* in both seasons. The data are consistent and indicate seasonal fluctuations in body mass and changes in the sex ratio in 1-year-old beetles, due to the entrance into their reproductive period.

Competing Interests: The authors have declared that no competing interests exist.

Introduction

The understory layer plays an important role in the proper functioning of forest ecosystems [1,2]. Furthermore, it serves as a rich source of food for herbivorous insects [3,4]. It is believed that the major reason for the high insect diversity and the high abundance of herbivores in this vegetation layer is that understory plants grow mostly in shade [5]. The majority of earlier studies have reported that leaves of plants growing in shade are more damaged by insects than sunlit ones, primarily because of lower levels of defence compounds [6,7,8,9]. Moreover, leaves of shaded plants are also thinner and less tough and as a result are more readily digestible [10,11]. Additionally, their surface is covered by a lower of structures that obstruct insect movement and grazing [12,13,14]. Thus, leaf chemistry and structure determine food quality and the resultant food preferences of folivores [15]. The selection of foraging sites by insects can be directly determined by light conditions and, consequently, temperature. A feeding preference for sunlit leaves is linked to a higher mobility and metabolic efficiency in ectotherms [16,17].

The species composition of the understory in European forests is composed of a number of shrubs. These include native shrubs in the Betulaceae and Rosaceae, including the widely distributed common hazel, *Corylus avellana* L. [18,19], as well as the native European bird cherry, *Prunus padus* L., [20,21] and the alien, invasive black cherry, *Prunus serotina* Ehrh. [22,23]. These species provide suitable food for herbivorous insects [21,24,25]. Shrubs of both these *Prunus* speciesstart their spring growth earlier than other shrubs and their leaves becomequickly and heavily infested by folivores [26], including beetles of the genus *Gonioctena*[27,28] and the bird-cherry ermine, *Yponomeuta evonymellus* L.[29,30]. In Central-Eastern Europe, the major folivore on both of the *Prunus* species is the polyphagousbroad-shouldered leaf beetle, *Gonioctenaquinquepunctata*Fabricius (Chrysomelidae;[28,31]). Within the natural geographical range of *C. avellana*, one of the most common folivores is the monophagousflea beetle, *Altica brevicollis coryletorum* (Chrysomelidae; [32,33]). Beetles of this species are frequently found on hazel shrubs and cause substantial defoliation [26].

The degree of leaf damage caused by insect herbivores strongly depends on the light conditions available for plant growth, or more specifically within a single shrub, whether the leaves are sunlit or shaded [26,34]. We have determined that the reduction in leaf mass caused by *G. quinquepunctata*, as well as by other species of folivores, in *P. padus* is much greater than in *P. serotina*. Shaded shrubs (under the canopy of trees)have a greater abundance of damaged leaves than those growing on sunny sites, irrespective of the *Prunus* species. In *P. serotina*, leaves of shaded shrubs are nearly exclusively eaten, although this species is much rarer in shaded sites due to its limited tolerance of low light conditions [35]. Depending on light conditions, the difference in the susceptibility of the two mentioned *Prunus*species to leaf damage by insect herbivores does not result from differences in the concentrations of defence compounds but rather primarily from differences in leaf structure, especially leaf toughness [26].

In contrast to the *Prunus* shrubs, sunlit leaves of*C. avellana* suffer much greater damage than shaded leaves [14,26]. The high degree of leaf damage on sunlit *C. avellana*is correlated with a higher abundance of larvae and adults of *A. brevicollis coryletorum*.

The present study was designed to determine how time of occurrence (understood as insect age—current-year in summer vs.1-year-old, in spring after winter diapause)affects body mass and the sex ratio (F/M) in both species in a natural environment. Additionally, how these relationships are affected by light conditions of host plant growth was examined. Among the many parameters that reflect more favourable living conditions for insects, e.g. shorter duration of larval development, increased potential fecundityand realizedfertility, and higher efficiency of conversion of ingested food [36,37], the most frequently recognized and easily measured

parameter is body mass [38,39]. The ratio of females to males(F/M ratio) may also be used to evaluate living conditions, preferences in the selection of favourable sites for offspring, and to explain differences in the body mass of beetles, depending on their age, before and during reproduction [40].

We hypothesized that a change in body mass in current-year insects would be determined only by the amount of consumed food. We further hypothesized that the F/M ratio in current-year insects should be stable, whereas in 1-year-old beetles, the F/M ratio should change due to reproduction. More specifically, we believed that in1-year-old insects (in the spring of the following year, after the winter diapause), females that had fulfilled their reproductive role would die after oviposition, while males would still be found on shrubs. If true, this would result in a successive decrease in the F/M ratio of 1-year-old beetles during time of their occurrence in spring. The observed differences in food preferences (sunlit vs. shaded leaves) of the studied species, *G. quinquepunctata* and *A. brevicollis coryletorum*, was also considered when evaluating our hypotheses. Lastly, we hypothesized thatall of the above-mentioned relationships would occur in both insect species. It was assumed that the F/M ratio would be similar in current-year beetles and would change in time in favour of 1-year-old males, irrespective of insect species and light conditions.

Materials and Methods

Plants

The study was carried out on understory shrubs ofblack cherry (*Prunus serotina* Ehrh.), European bird cherry (*P. padus* L.), and common hazel (*Corylus avellana* L.). Field research was conducted in a permanent plot in Tulce (Kobylepole Forest; Babki Forest District; Poland; 52° 36' N, 17°06' E). Shrubs utilized in the study were 3-5-m high, growing either in high light (full light for a few hours per day) orshaded conditions (about 15–30% of full light), under the canopy of *Pinus sylvestris* L. with an admixture of *Quercusrobur* L., *Fagussylvatica* L., *Carpinusbetulus* L., and *Ulmuslaevis* Pall. Light intensity in the crowns of selected shrubs was measured with a LAI-2200 Plant Canopy Analyzer. A total of 48 shrubs of *P. padus* and48 shrubs of *P. serotina*(24 sunlit and 24shaded shrubs of each species)were marked and utilized, as well as80 shrubs of *C. avellana*(40 shrubs in each light condition).

Insect species and field research

The study utilizedthe polyphagous beetle, *Gonioctenaquinquepunctata*Fabricius 1787 (syn. *Phytodectaquinquepunctata*, Kirby 1837), and the monophagousbeetle, *Alticabrevicolliscoryletorum*Král 1964 (Coleoptera, Chrysomelidae; [41,42]), both of which have a similar life cycle (current-year adults emerge in summer, and only in early spring, after the winter diapause, the 1-year-old beetles reproduce).*A.brevicollis coryletorum*is characterized by distinct food preferences, determined by light conditions of host plant growth, i.e. it feeds primarily on sunlit leaves. In contrast, *G.quinquepunctata*, prefers leaves of shaded shrubs. Only adult insects were examined in the present study.

This study was carried out in strict accordance with the ethical standards in entomological research. The studied species are one of the major pests of black cherry, European bird cherry, andcommon hazel in Poland. In addition, the collection of insects in Poland is allowed without additional permission due to the public access to the forest and opportunities to insects, fruits, and mushrooms collection.

Adult *G. quinquepunctata* were captured in two seasons: in July, 2013 (current-year) and in May, 2014 (1-year-old, after the winter diapause). After the appearance of the first insects on leaves (01.07.2013 for current-year and 02.05.2014 for 1-year-old beetles) on the marked

shrubsof *Prunus padus* and *P. serotina*, three shrubs of each light variant were randomly selected and beetles present on the shrubs were collected using an entomological umbrella. Each shrub was used only once in order to avoid the negative effect of an earlier disturbance on insect collection. Current-year *G. quinquepunctata* beetles were observed in the field for 15 days, whereas after the winter diapause, 1-year-old beetles were observed for 21 days. For each variant of the experiment, the number of insects was approximately 15–30/shrub. When the number of insects on a shrub was <10, they were supplemented with insects from a neighbouring shrub.

In the case of *A. brevicollis coryletorum*, beetles were collected in August and early September, 2013 (current-year) and in April and May, 2014 (1-year-old, after the winter diapause). After the appearance of the first insects on leaves (02.08.2013 for current-year and 11.04.2014 for 1-year old beetles) on shrubs of *Corylus avellana*, four shrubs of each light variant were randomly selected every six days, and approximately 15–30 individual insects were collected from each shrub. Current-year beetles of *A. brevicollis coryletorum*were observed in the field for 36 days, although single individuals were also found in late September, whereas after the winter diapause, 1-year-old beetles were collected for 54 days.

The captured beetles of both species were killed with ethyl acetate and their mass and sex was recorded about 18 h later. Body mass was measured with an analytical balance to the nearest 0.01 mg (Sartorius CP225D), while sex was determined under a stereomicroscope. In the case of *A. brevicollis*, *coryletorum*, the genitals were also exposed and examined with the use of microsurgical instruments.

Statistical analyses

Analysis of variance (ANOVA)was used to determine the effect of shrub species (for *G. quinquepunctata*), light conditions, sex, and age on beetle body mass and the F/Mratio, inboth insect species. During the analysis, the factor "term" (defined as length of the sampling period) was nested in the factor "age" (term[age]; understood astime of occurrence of insects in summer, and in spring, after the winter diapause). Additionally, a one-way ANOVA was used to analyse differences in the F/M ratio (for *G. quinquepunctata*) and beetle mass (for *A. brevicollis coryletorum*). Shrubs were treated as a random factor. A Tukey's HSD test was used to determine significant differences between treatments when necessary. Relationships between the collection term and mass, as well as the F/M ratio, were examined and displayed as regression equations. The curves presented in the figures were generated on the basis of regression equations in whichR^2 was the highest and statistically significant. All analyses were conducted using JMP 8 software (SAS Institute, Cary, NC, USA).

Results

Gonioctena quinquepunctata

A significant effect of insect age on the body mass of *G. quinquepunctata* was observed. Average mass of 1-year-old beetles caught in spring (after winter diapause) were2.6% heavier than current-year beetles in summer (Table 1). Additionally, the significant age × sex interaction indicated that the differences in body mass in females between seasons were significant (in spring they were heavier), while no significant differences or interactions were detected in males. The time of collection significantly affected body mass, both in summer (current-year beetles) and in the spring of the following year (1-year-old beetles; Table 1). In both seasons, a significant effect of sex on body mass was observed, as was a significant sex × term interaction. In current-year beetles, the body mass of both females and males markedly increased on successive collection dates (Fig 1A). Initially, the F/M ratio was equal to 1. It subsequently

Table 1. Summary of ANOVA on the effectsoftime of occurrence(age: current-yearvs1-year-old), cherry species (*PrunuspadusvsP. serotina*), light conditions (highlight vs shade), sex (female vs male), term(length of the sampling period, see Materials and Methods) and their interactions on the body mass of adult *Gonioctena quinquepunctata*beetles. The factor "term" was nested in the factor "age" (term[age]).*P* values<0.05 are in bold.

ANOVA	Beetle body mass (mg)		
	d.f.	F	P
Age	1	130.4694	**<0.0001**
Species	1	15.7971	**<0.0001**
Light	1	0.7548	0.3850
Sex	1	6895.319	**<0.0001**
Term[age]	12	44.9844	**<0.0001**
Age×species	1	16.8919	**<0.0001**
Age×light	1	1.0695	0.3011
Age×sex	1	14.1207	**0.0002**
Species×light	1	5.1939	**0.0227**
Species×sex	1	2.9488	0.0860
Light×sex	1	0.0564	0.8122
Light×term[age]	12	5.2843	**<0.0001**
Sex×term[age]	12	3.6386	**<0.0001**
Age×species×light	1	4.7394	**0.0296**
Species×term[age]	12	3.0855	**0.0002**
Age×species×sex	1	7.2482	**0.0071**
Age×light×sex	1	0.0181	0.8929
Species×light×sex	1	0.0185	0.8917
Species×light×term[age]	12	2.9468	**0.0004**
Species×sex×term[age]	12	2.4201	**0.0040**
Light×sex×term[age]	12	1.5531	0.0983
Age×species×light×sex	1	0.1226	0.7263
Species×light×sex×term[age]	12	1.4413	0.1395
Errord.f.		3238	

doi:10.1371/journal.pone.0144718.t001

increased over time and then slightly declined by the end of the season. Females, however, still outnumbered males (Fig 1A). In contrast, the F/M ratio in 1-year-old beetles decreased (Fig 1B). While male body mass increased over time, it remained stable in females (Fig 1B). The average insect body mass of both age groups feeding on *P. padus* were significantly heavier than those feeding on leaves of *P. serotina*. The difference, however, reached only about 1.7%. Notably, among the insects feeding in the summer (current-year), beetles feeding on leaves of *P. padus* were 5.8% heavier than those collected from shrubs of *P. serotina* (Fig 2). Light conditions for the shrubs, however, did not significantly affect the body mass of the beetles.

Results further indicated a significant effect of age, light conditions, collection term, and an age × species interaction on F/M ratio (Table 2). The F/M ratio of current-year beetles was 22.1% higher on shrubs of *P. padus* than on shrubs of *P. serotina*, and 30.4% higher on sunlit shrubs than on shaded shrubs (Fig 3A and 3B). In 1-year-old beetles, the F/M ratio was 23.5% higher on *P. serotina* than on *P. padus* (Fig 3C), and 38.5% higher on sunlit shrubs than on shaded shrubs(Fig 3D).

Altica brevicollis coryletorum

A significant effect of insect age on average body mass was observed in both female and male beetles.1-year-old beetles weighed over 25% more than those caught in summer in the year of

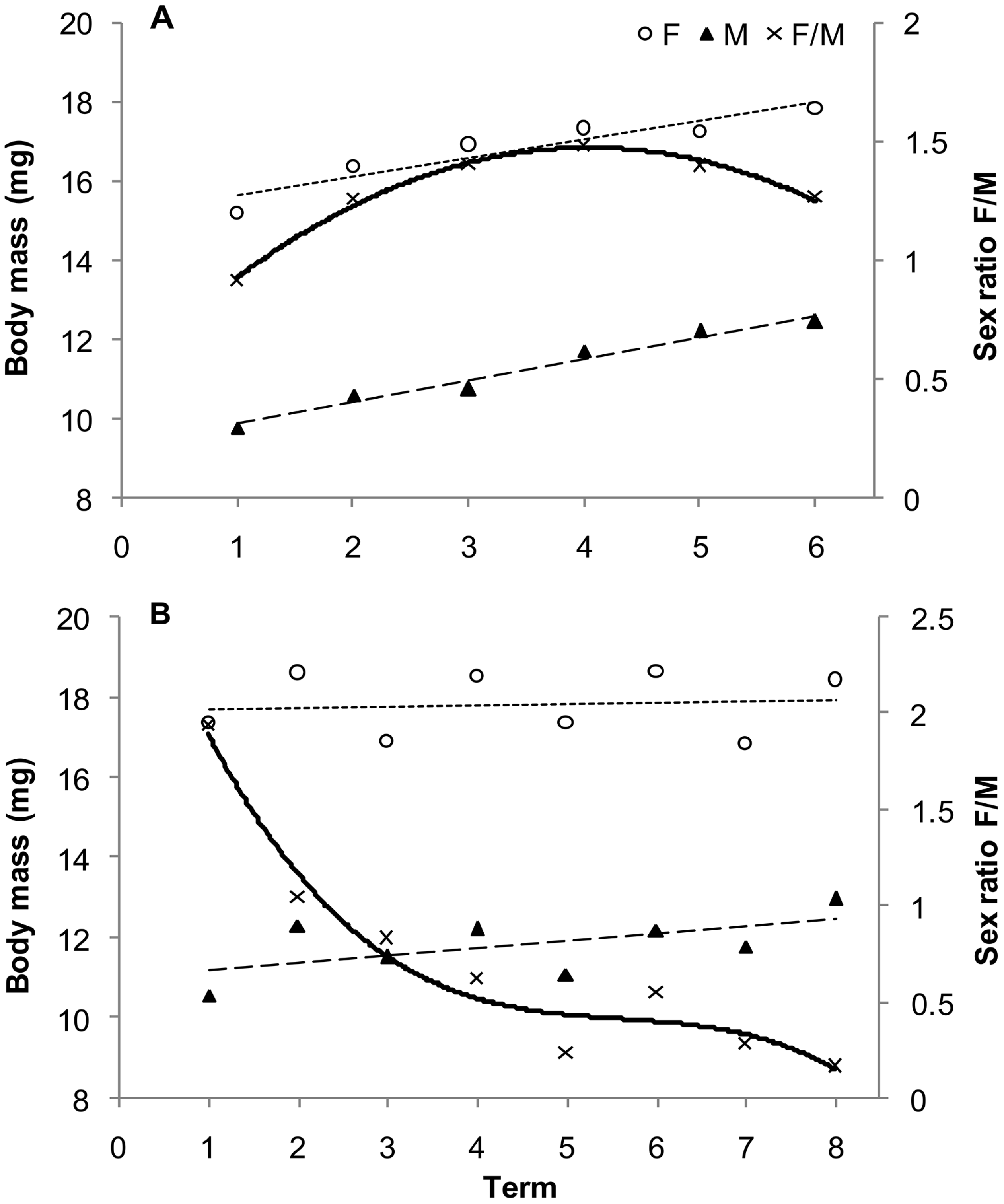

Fig 1. Body mass and sex ratio of current-year (A) and 1-year-old (B) *Gonioctena quinquepunctata*beetles over time. Numbers 1–6 and 1–8 indicate successive datesof insect collection (see Materials and Methods). One-way ANOVA was used to determine the effect of term (length of the sampling period) on the body mass of females (current-year,$R^2 = 0.1177$, $P<0.0001$; 1-year-old,$R^2 = 0.0895$, $P<0.0001$), males (current-year,$R^2 = 0.2902$, $P<0.0001$; 1-year-old,$R^2 = 0.1882$, P<0.0001), and the sex ratio(current-year,$R^2 = 0.1284$, $P = 0.0986$; 1-year-old,$R^2 = 0.7117$, $P<0.0001$).

doi:10.1371/journal.pone.0144718.g001

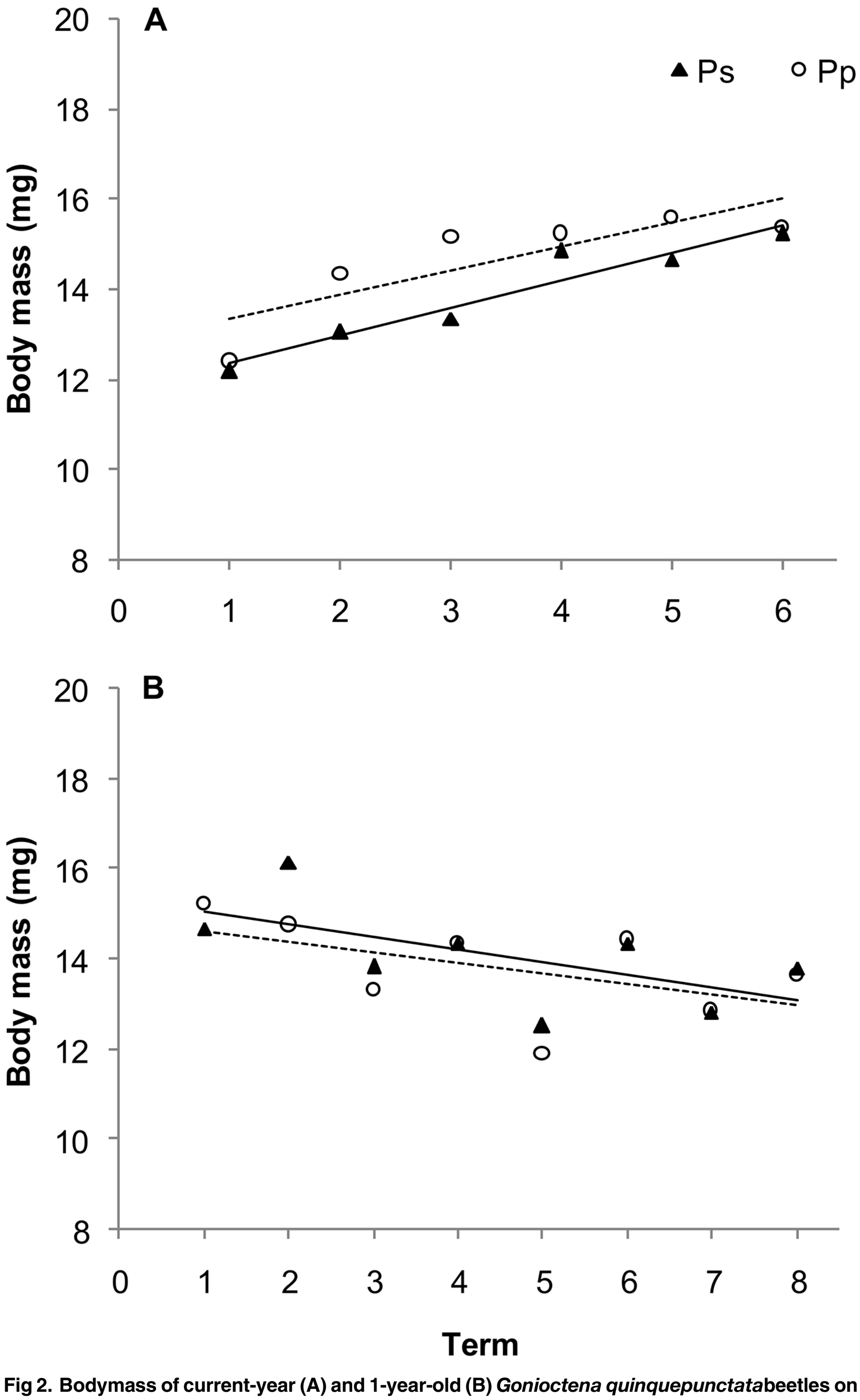

Fig 2. Bodymass of current-year (A) and 1-year-old (B) *Gonioctena quinquepunctata*beetles on *Prunus padus* (Pp) and *Prunus serotina* (Ps) shrubs over time. Numbers 1–6 and 1–8 indicate successive dates of insect collection (see Materials and Methods). One-way ANOVA was used to determine the effect of term (length of the sampling period)on the body mass of beetles on Ps (current-year,R^2 = 0.0999, P<0.0001; 1-year-old,R^2 = 0.0894, P<0.0001) and on Pp (current-year,R^2 = 0.0878, P<0.0001; 1-year-old,R^2 = 0.1005, P<0.0001).

doi:10.1371/journal.pone.0144718.g002

Table 2. Summary of ANOVA on the effectsoftime of occurrence(age: current-yearvs1-year-old), cherry species (*PrunuspadusvsP. serotina*), light conditions (highlight vs shade), term(length of the sampling period, see Materials and Methods) and their interactions on the F/M sex ratio of adult *Gonioctena quinquepunctata*beetles. The factor "term" was nested in the factor "age" (term[age]). *P* values <0.05 are in bold.

ANOVA	Sex Ratio (F/M)		
	d.f.	F	P
Age	1	106.2627	**<0.0001**
Species	1	0.4542	0.5017
Light	1	21.3320	**<0.0001**
Term[age]	12	18.5652	**<0.0001**
Age×species	1	11.2054	**0.0011**
Age×light	1	1.3030	0.2561
Species×light	1	0.6337	0.4277
Light×term[age]	12	1.7699	0.0617
Age×species×light	1	0.0262	0.8716
Species×term[age]	12	1.1359	0.3388
Species×light×term[age]	12	1.5637	0.1125
Errord.f.		112	

doi:10.1371/journal.pone.0144718.t002

their emergence (Table 3). Moreover, a significant age × sex interaction was also observed, with a descending order of body mass as follows:1-year-old females (a), current-year females (b), 1-year-old males (c) and current-year males (d) (Tukey's HSD test; $P<0.05$).

In beetles feeding in the current year, the body mass of females and males increased over successive collection dates (Fig 4A), but the increases were smaller in males than in females. In1-year-old beetles, body mass of both female and male beetles increased overtime, but initially increased more rapidly in females and then became more stable (Fig 4B). Values of the F/M ratiowere lower than 1 throughout both of the seasons of occurrence(Table 4). In 1-year-old the F/M ratio also changed significantly over the term of collection. It initially rose very rapidly and then fell dramatically over the term of the collection period (Fig 4B).

A significant effect of light conditions, sex, and term on body mass was observed in both seasons (Table 3). The body mass of current-year (Fig 5A) and1-year-old beetles (Fig 5B) feeding on sunlit shrubs was significantly higher than in beetles collected from shaded shrubs. Difference reached as high as14.8% ($F_{1,954} = 100.89$, $P<0.0001$)in current year beetles and 13.7% ($F_{1,1669} = 80.50$, $P<0.0001$) in 1-year-old beetles. All of the examined factors (age, light conditions, and collection term)significantly affected the F/M ratio in both current-year and 1-year-old beetles (Table 4). The light × term interaction was also significant (Fig 6). The F/M ratio incurrent-year beetles was 18% higher (Fig 6A)than in1-year-old beetles. Interestingly, the F/M ratioin1-year-old beetles collected from sunlit shrubs was 28.2% higherthan in beetles collected from shaded shrubs. The timing of peaks in the F/M ratio also differed between the two groups (Fig 6B).

Discussion

In the present study, we observed a significant effect of sex and collection term on the body mass in both species of beetles and in both foraging seasons (current-year and 1-year-old beetles). In general, females of most insect species are much heavier than their male counterparts [43,44]. This observation was also determined to be true for female beetles in earlier studies of *Gonioctena quinquepunctata*[45]and *Altica brevicollis coryletorum*[14]. Changes in the body mass of current-year beetles of both sexes occurred as a direct result of the amount of feeding.

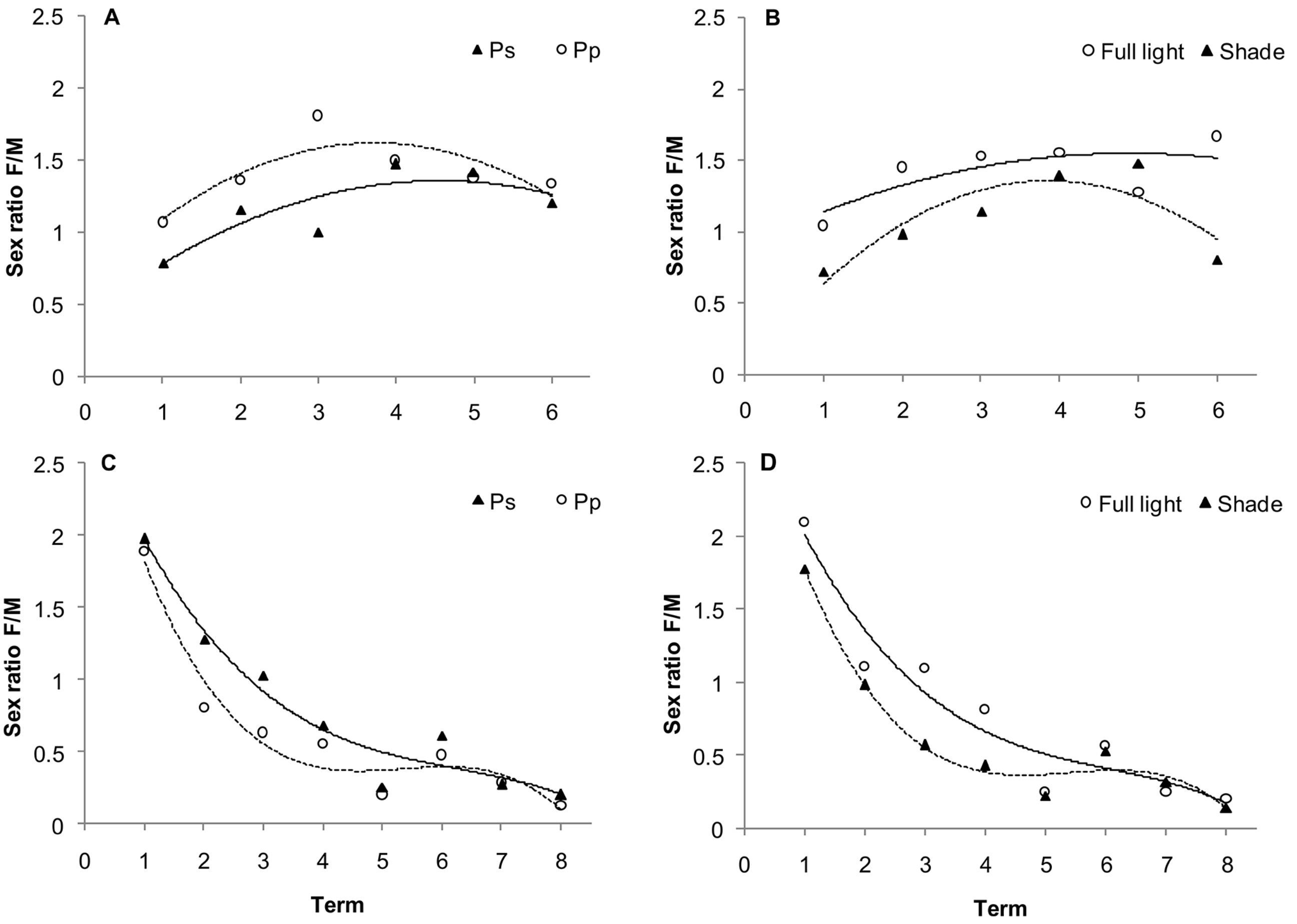

Fig 3. Sex ratioof current-year (A and B) and 1-year-old (C and D) *Gonioctena quinquepunctata*beetles on *Prunus padus* (Pp) and *Prunus serotina* (Ps) shrubs growing in full light or shade(B and D). Numbers1-6 and 1–8 indicate successive dates of insect collection (see Materials and Methods). One-way ANOVA was used to determine the effectof term(length of the sampling period)on the sex ratio of beetles on Ps (current-year,R^2 = 0.2477, P = 0.1111; 1-year-old,R^2 = 0.7098, P<0.0001), on Pp (current-year,R^2 = 0.1884, P = 0.2551; 1-year-old,R^2 = 0.7661, P<0.0001), on sunlit shrubs (current-year,R^2 = 0.1286, P = 0.5031; 1-year-old,R^2 = 0.8441, P<0.0001) and shaded shrubs (current-year,R^2 = 0.4109, P = 0.0053; 1-year-old,R^2 = 0.8590, P<0.0001).

doi:10.1371/journal.pone.0144718.g003

An important role was also played by other factors in the spring following diapauses. These factors were linked to reproduction and the post-reproductive decline in population size. Females in the genus *Gonioctena*[42]and in the species *Altica brevicollis coryletorum*, hide in leaf litter or die, while males remain active on shrub leaves. The observation that the sex ratio of a population changes with time of occurrence is simply the result of sex-specific difference in age-related mortality, with female that fulfilled reproduction dying earlier than males. Female-biased mortality in 1-year-oldbeetlesmay be caused by an extensive energy expenditure in short time or different patterns of behaviour after reproduction in relation to the male. There were no indications of parental care, although such behaviour has been previously reported for other species in the Chrysomelidae family [46]. In the present study, we observed the coexistence of unfertilized and fertilized females on leaves of the selected host plants, along with females that had already laid their eggs and remained on the host leaves for some time after oviposition. The presence of so many different types of females would undoubtedly affect the mean body mass of females in a given sample. Moreover female-biased mortality in

Table 3. Summary of ANOVA on the effects oftime of occurrence(age: current-yearvs1-year-old), light conditions (high light vs shade), sex (female vs male), term(length of the sampling period, see Materials and Methods) and their interactions on the body mass of adult*Altica brevicollis coryletorum* beetles. The factor "term" was nested in the factor "age" (term[age]). *P* values <0.05 are in bold.

	Body mass (mg)		
ANOVA	d.f.	F	P
Age	1	308.1319	**<0.0001**
Light	1	181.4572	**<0.0001**
Sex	1	2541.049	**<0.0001**
Term[age]	15	29.0078	**<0.0001**
Age×light	1	5.2781	**0.0217**
Light×sex	1	6.2855	**0.0122**
Age×sex	1	172.1252	**<0.0001**
Light×term[age]	15	5.0669	**<0.0001**
Sex×term[age]	6	8.4715	**<0.0001**
Age×sex×light	1	0.5434	0.4611
Term× light×sex[age]	6	0.5213	0.9305
Errord.f.		2559	

doi:10.1371/journal.pone.0144718.t003

reproduction season could be understood as an altruistic behaviour or specific "parental care", when females reduce competition for food for their offspring and hide in litter or die quickly.

Results of our study suggest that beetles of both species foraging in spring (1-year-old)are heavier than those collected in summer, which may be attributed to differences in food quality. Leaf structure and chemistry change with leaf age. Older leaves contain less nitrogen [47], non-structural carbohydrates and water [48,49], while concentrations of secondary metabolites increase [26,50]. The structure of leaves also changes with age, especially during the later stages of development. Leaves become tougher, more leathery, and theoretically more difficult for folivores to ingest and digest [14,51]. The described developmental and structural changes suggest that younger leaves are a qualitatively more nutritious and edible food source for most insect folivores. Moreover, beetles are involved in reproductive processes during the spring months when in the summer months are only focused on foraging for suitable food and defence against unfavourable environmental factors [42]. Thus, the efficiency of utilizing young leaves must be much higher than in summer. This was most conspicuous in 1-year-old beetles of *A. brevicollis coryletorum*, whose mass, despite the energy loss for reproduction, was 25% higher than in current-year beetles before winter.

The relatively high level of defence compounds (e.g. soluble phenolics and tannins) present in leaves of both *Prunus* species apparently does not provide a sufficient defence against herbivory by foliovores [45,52]. Therefore, they are greatly damaged by various species of insect herbivores, and in particular by the polyphagous, *G. quinquepunctata*[26,28]. Our present data on the effect of *Prunus* species on the body mass of adult *G. quinquepunctata* beetles are consistent with the results of our earlier study [45], which indicated that only small differences in leaf chemistry are present in the leaves of these species in the seasons when beetles are searching for food. The effect of leaf source on body mass is more evident in current-year beetles when body mass differed by approximately6% (Fig 2A) between the two food sources. At a later stage of the growing season, leaves of *P. serotina*are tougher and more leathery. Consequently, a large investment in chemical defence against folivores is not required [10,23,30]. Moreover, *P. serotina* is highly resistant to a variety of biotic factors that reduce leaf area [28]and to mechanical damage; in addition to partial or even complete defoliation [53,54]. In the present study,

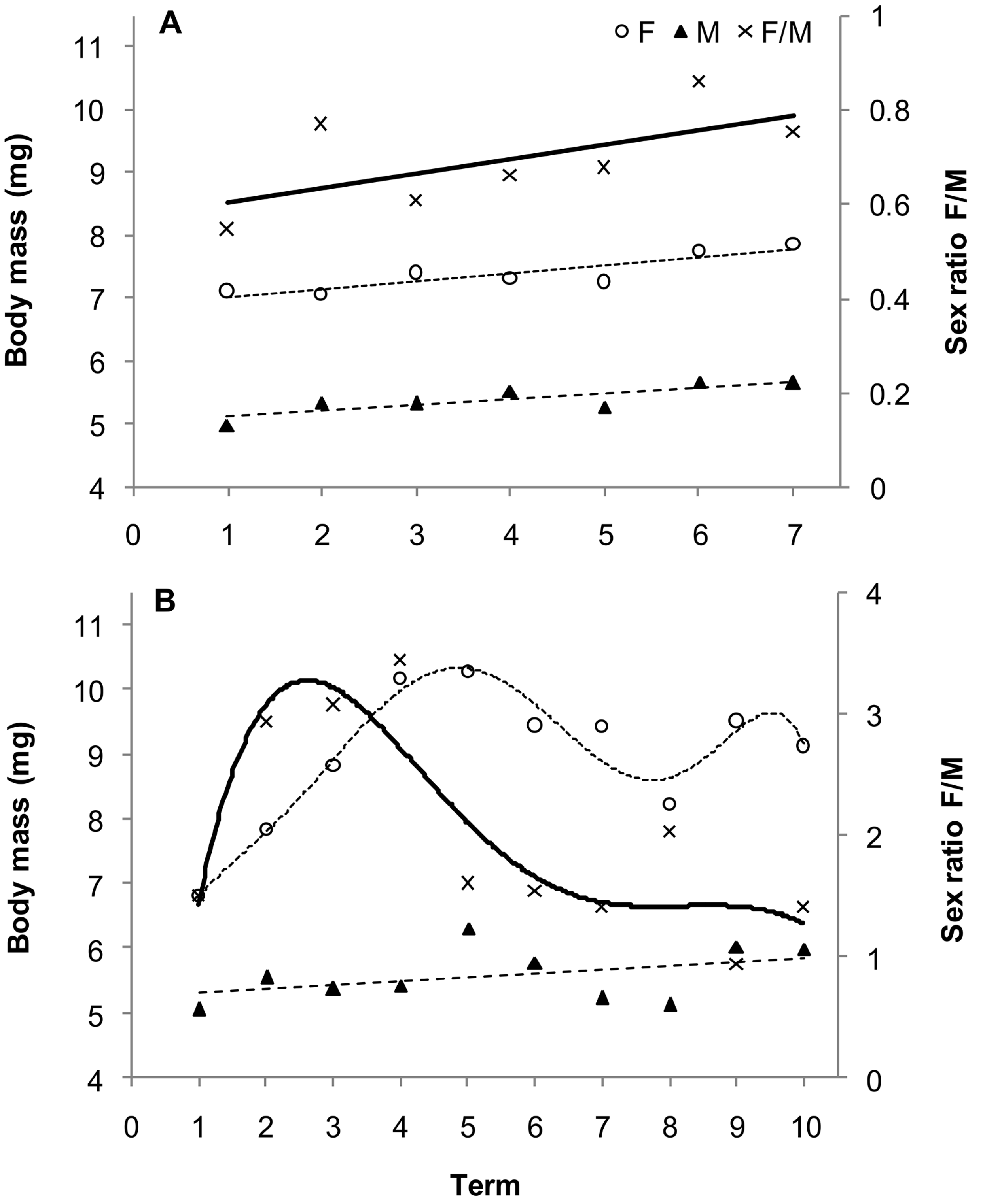

Fig 4. Body mass and sex ratio of current-year (A) and 1-year-old (B) *Altica brevicollis coryletorum* beetles over time. Numbers 1–7 and 1–10 indicate successive dates of insect collection (see Materials and Methods). One-way ANOVA was used to determine the effectof termon females (current-year,R^2 = 0.0824, P<0.0001; 1-year-old,R^2 = 0.2530, P<0.0001), males (current-year,R^2 = 0.0726, P<0.0001; 1-year-old,R^2 = 0.1393, P<0.0001), and the sex ratio(current-year,R^2 = 0.1028, P = 0.4779; 1-year-old,R^2 = 0.1545, P = 0.1955).

doi:10.1371/journal.pone.0144718.g004

Table 4. Summary of ANOVA on the effectsoftime of occurrence(age: current-yearvs1-year-old), light conditions (highlight vs shade), term(length of the sampling period, see Materials and Methods) and their interactions on the F/M sex ratio of adult *Altica brevicollis coryletorum* beetles. The factor "term" was nested in the factor "age" (term[age]). *P* values <0.05 are in bold.

	Sex ratio (F/M)		
ANOVA	d.f.	F	P
Age	1	121.7511	**<0.0001**
Light	1	10.2660	**0.0018**
Term[age]	15	6.4989	**<0.0001**
Age×light	1	3.8466	0.0526
Light×term[age]	15	5.8376	**<0.0001**
Errord.f.		100	

doi:10.1371/journal.pone.0144718.t004

the small difference observed in the body mass of beetles feeding on different *Prunus* species indicates that leaves of both *Prunus* species are both similar in their quality as a food source for *G. quinquepunctata*.

Our data indicate that in the wild, the better light conditions present forleaves of *Corylus avellana*, which are eaten by larvae and adults of *A brevicollis coryletorum*, produce a food source that results in a higher body mass in beetles in both foraging seasons (summer and spring of the following year, Table 3). These results are consistent with the results of our earlier laboratory experiments, where the possibility to choose a food source and the effects of other environmental factors were not present [14]. Many studies indicate that strong light causes slower insect growth and development [7,9,55]. In some insect species, however, sunlit leaves are more favourable than shaded leaves for insect growth and development [17,56]. In relative comparison to shaded leaves, sunlit leaves of *C. avellana*contain higher concentrations of defence compounds, such as phenolics and condensed tannins [26], and are characterized by greater toughness and density of trichomes and glandular trichomes containing phenols [14]. In the case of *C. avellana*, however, these factors do not provide a sufficient defence system against the leaf-eating beetle, *A. brevicollis coryletorum*. This insect species is a typical example of monophagy, and such specialist insects often tolerate higher levels of repellents than generalist species do [57]. Additionally, feeding on sunlit leaves which are rich in non-structural carbohydrates, results ina significant increase in body mass and a shortening of insect development [58]. Moreover, the higher quality food source, along with the higher temperatures present in sunlit leaves, provides an effective mechanism of defence of this beetle species against predators and allows them to jump over larger distances [14].

In both insect species, different trends in changes of the body mass of females and males, as well as in the F/M ratio overtime depending on insectage, are a result of the different biology of females at various stages of their life cycle. This is reflected in the relatively stable F/M ratio of current year beetles in the first summer (Figs 1A and 4A). In contrast, females appear earlier and initially prevail during spring of the following spring, but they die sometime after oviposition. Males, however, are more numerous and exhibit a relatively stable and low F/M ration by the end of the season (Figs 1B and 4B). It should be emphasized that the number of females and males at the time of occurrence of 1-year-old insects are quite similar, but there is only a sex-specific shift at the time of their occurrence. Data from the present study indicate a significant effect of light conditions (sun vs. shade) on shrub growth andage × host species interaction on the F/M ratio in *G. quinquepunctata* (Table 2). Food quality may also affect various components of the reproductive strategy of herbivorous insects, e.g. resource allocation to eggs, egg size and quality, and sex ratios [15]. The higher abundance of current-year females on *P. padus*

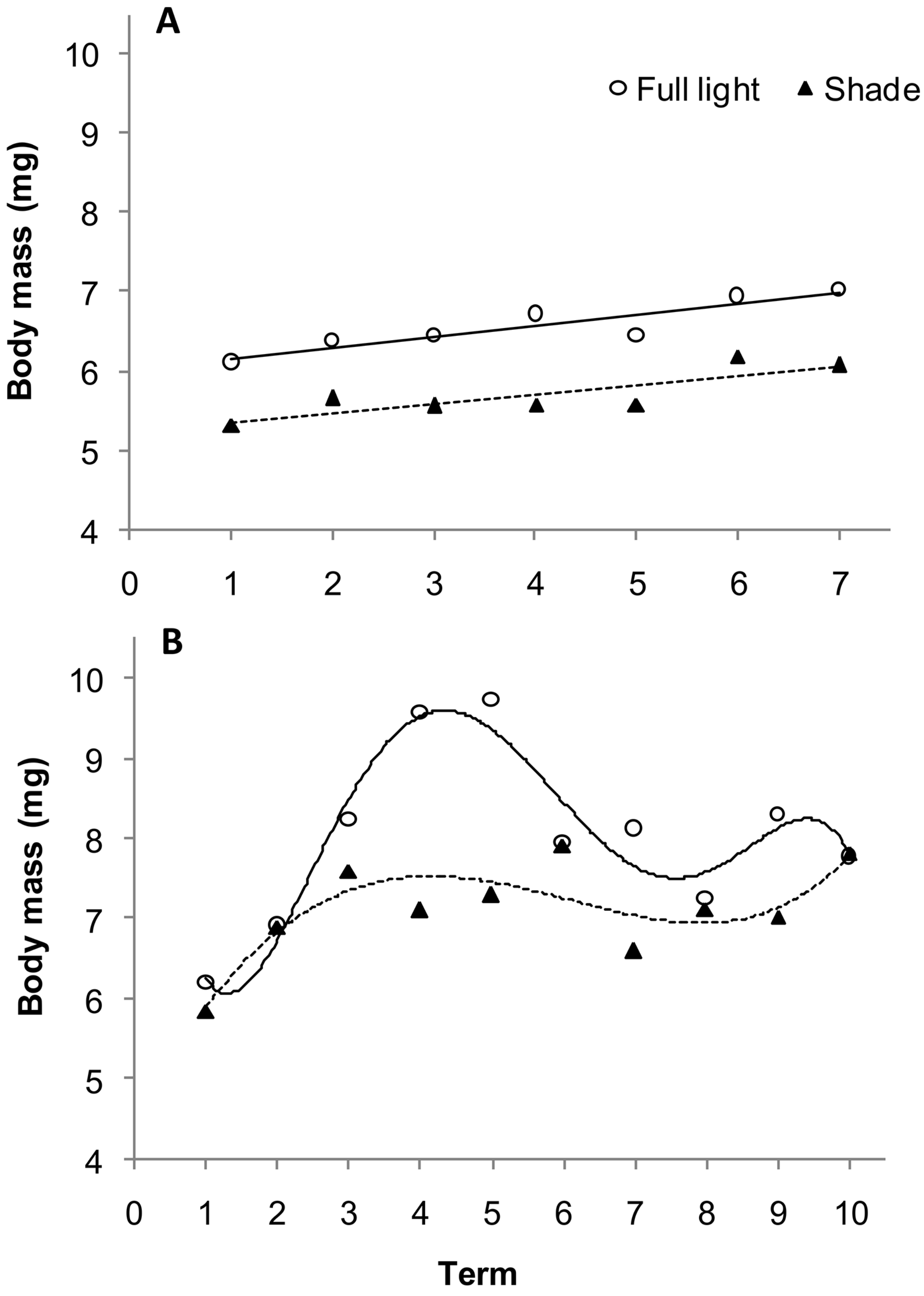

Fig 5. Body mass of current-year (A) and 1-year-old (B) *Altica brevicollis coryletorum*beetles on shrubs growing in full light and shaded conditions over time. Numbers 1–7 (for current-year beetles) and 1–10 (for 1-year-old beetles) indicate successive dates of insect collection (see Materials and Methods). One-way ANOVA was used to determine the effectof term(length of the sampling period) on current-year beetles (full light, $R^2 = 0.0515$, $P<0.0001$; shade, $R^2 = 0.0576$, $P = 0.0002$) and on1-year-old beetles (full light, $R^2 = 0.1742$, $P<0.0001$; shade, $R^2 = 0.0616$, $P<0.0001$).

doi:10.1371/journal.pone.0144718.g005

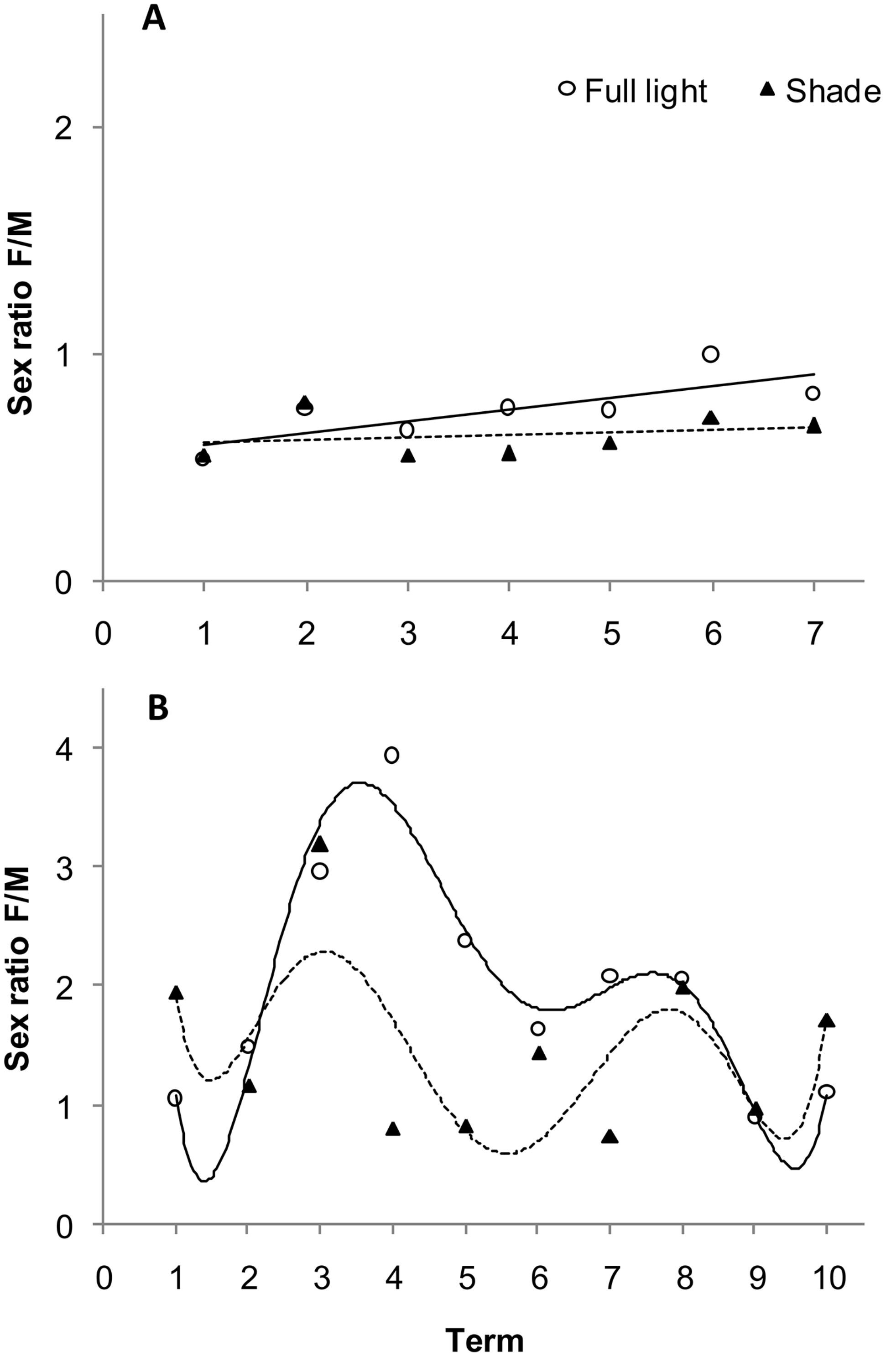

Fig 6. Sex ratio of current-year (A) and 1-year-old (B) *Altica brevicollis coryletorum*beetles on shrubs growing in full light and shaded conditions over time. Numbers 1–7 (for current-year) and 1–10 (for 1-year-old) indicate successive dates of insect collection (see Materials and Methods). One-way ANOVA was used to determine the effectof term (length of the sampling period)on current-year beetles (full light, R^2 = 0.2106, P = 0.4918; shade, R^2 = 0.0754, P = 0.9374) and 1-year-old beetles (full light, R^2 = 0.5475, P = 0.0018; shade, R^2 = 0.2715, P = 0.3076).

doi:10.1371/journal.pone.0144718.g006

(higher F/M ratio) and the greater abundance of1-year-oldfemales on*P*. *serotina*may be the result of higher food quality or better living conditions represented by the leaves of these species and their access to light. It is also plausible that a preference for sunlit shrubs by both age groups is also a contributing factor as well. In full light, higher temperatures and lower competition create more favourable conditions for female *G*. *quinquepunctata* beetles in regards to offspring allocation. In support of this premise, Wennström et al. [59], in their research on food preferences of *G.linnaeana*, reported that host plant selection by females is determined by higher food quality. The authors suggested that *G*. *linnaeana* females have evolved a behaviour that maximizes offspring performance and thus positively influences female fitness when feeding on a preferred food source. There are several plausible reasons for the existence of a relationship between host selection and offspring efficiency. Potential factors that would have a large impact on the aforementioned relationship include: female food preferences, differences in female investment in eggs, and ensuring the safety of the offspring by the female [40,59,60]. We hypothesize that the higher F/M ratio on sunlit shrubs for both of the studied host species may be the result of optimum food source selection for adults of the next generation over the selection of the lower quality food source represented by leaves on shaded shrubs. Additionally, herbivores can benefit from the dispersal of alien, newly-invasive plants, as more food becomes subsequently available. Occupation of new plant species makes it possible to avoid or reduce the exposure of insects to parasites and predatory attacks [61]. This may explain, to a large extent, the elevated F/M ratio in *G*. *quinquepunctata* on the alien, newly-invasive shrub, *P*. *serotina*, during insect reproduction (Fig 3C). This premise is supported by the results of our earlier field research, where the body mass of beetles was higher when they fed on sunlit shrubs of *P*. *serotina* [45].

In contrast to experiments where larvae cannot choose their food source, insects studied in natural environments are affected by a complex of multiple factors including the availability of high quality food, leaf structure and chemistry [15], and intra-and interspecific competition [62,63]. In a natural environment, the presence of predators [39]and parasites [64], specific behaviour [59], as well as phenological differences between host plants [30], can play a major role on the biology of an insect. In general, the data obtained in the present study are similar for both of the studied beetle species. In summer, after the eggs hatch into larvae, the body mass of both male and female adult beetles increases. During that period, the F/M ratio is relatively stable. In the following spring after the winter diapause, the F/M ratio changes dramatically, primarily due to the disappearance of females at a period of time after oviposition. Importantly, we found that the length of the period of reproduction, as well as female and male behaviour in insect populations, significantly influences body mass. The results of the present study contribute to confirming the pattern and complexity of these changes. Taking into account the different adaptations of males and females to their distinct reproductive roles, seasonal fluctuations in body mass and the F/M ratio occur in relation to the reproductive cycle. We believe that additional research on other species in the Chrysomelidae, or in other insect families possessing species that have a similar life cycle, will confirm our conclusions.

Acknowledgments

We are grateful to the Babki Forest District for allowing us to conduct field research.

Author Contributions

Conceived and designed the experiments: AŁ PK. Performed the experiments: AŁ EM. Analyzed the data: AŁ EM MJG PK. Contributed reagents/materials/analysis tools: AŁ EM. Wrote the paper: AŁ EM MJG PK.

References

1. Crawley MJ. The structure of plant communities. In: Crawley MJ, editor. Plant Ecology. London: Blackwell; 1986. pp. 1–50.
2. Decocq G, Aubert M, Dupont F, Alard D, Saguez R, Wattez-Franger A, et al. Plant diversity in a managed temperate deciduous forest:understorey response to two silvicultural systems. J Appl Ecol. 2004; 41: 1065–1079.
3. Leather SR.Medium term effects of early season defoliation on the colonisation of bird cherry (*Prunus padus*) by insect herbivores. Eur JEntomol.1995; 92(4): 623–631.
4. Hamilton WT, McGinty A, Ueckert DN, Hanselka CW, Lee MR. Brush Management: Past, Present, Future. Texas: A&M University Press; 2004.
5. Southwood TRE. The number of species of insect associated with various trees. J Anim Ecol. 1996; 30 (1): 1–8.
6. Crone EE, Jones CG. The dynamics of carbon-nutrient balance: effects of cottonwood acclimation to short- and long-term shade on beetle feeding preferences. J Chem Ecol. 1999; 25(3): 635–656.
7. Henriksson J, Haukioja E, Ossipov V, Osipova S, Sillsnpää S, Kaspari L, et al.Effects of host shading on consumption and growth of the geometrid *Epirritaautumnata*: interactive roles of water, primary and secondary compounds.Oikos. 2003; 103: 3–16.
8. Koricheva J. Nykänen H, Gianoli E. Meta-analysis of trade-offs among plant antiherbivore defenses: are plants jacks-of-all-trade, masters of all? Am Nat. 2004; 163: E64–E75. PMID: 15122510
9. Roberts MR, Paul ND. Seduced by the dark side: integrating molecular and ecological perspectives on the influence of light on plant defence against pests and pathogens. New Phytol. 2006; 170: 677–699. PMID: 16684231
10. Abrams MD, Kloeppel BD, Kubiske ME.Ecophysiological and morphological responses to shade and drought in two contrasting ecotypes of *Prunus serotina*. Tree Physiol. 1992; 10: 343–355. PMID: 14969972
11. Dominy J, Grubb PJ, Jackson RV, Lucas PW, Metcalfe DJ, Svenning J-C, et al. In tropical lowland rain forests monocots have tougher leaves than dicots, and include a new kind of tough leaf. Ann Bot.2008; 101: 1363–1377. doi: 10.1093/aob/mcn046 PMID: 18387969
12. Hardin JW, Murrell ZE. Foliar micromorphology of *Cornus*. J Torrey Bot Soc. 1997; 124(2): 124–139.
13. Molina-Montenegro MA, Ávila P, Hurtado R, Valdivia AI, Gianoli E. Leaf trichome density may explain herbivory patterns of *Actinote sp*. (Lepidoptera: Acraeidae) on *Liabummandonii* (Asteraceae) in a montane humid forest (Nor Yungas, Bolivia). ActaOecol.2006; 30: 147–150.
14. Łukowski A, Giertych MJ, Zadworny M, Mucha J, Karolewski P. Preferential feeding and occupation of sunlit leaves favorsdefense response and development in the flea beetle, *Altica brevicollis coryletorum* —a pest of *Corylus avellana*. PLOS ONE. 2015; 10(4): e0126072. doi: 10.1371/journal.pone.0126072 PMID: 25927706
15. Awmack CS, Leather SR. Host plant quality and fecundity in herbivorous insects. Annu Rev Entomol.2002; 47(1): 817–844.
16. Jaworski T, Hilszczański J. The effect of temperature and humidity changes on insects development their impact on forest ecosystems in the expected climate change. Leś Pr Bad. 2013; 74(4): 345–355.
17. Łukowski A, Mąderek E, Karolewski P.Influence of light conditions on bird cherry ermine moth—the main pest of bird cherry. Sylwan. 2015; 158(8): 595–603.
18. Deacon J. The location of refugia of *Corylusavellana* L. during the Weichselian glaciation. New Phytol. 1974; 73(5): 1055–1063.
19. Gökirmak T, Mehlenbacher SA, Bassil NV. Characterization of European hazelnut (*Corylus avellana*) cultivars using SSR markers.Genet Resour Crop Evol. 2009; 56(2): 147–172.
20. Leather SR.Prunus padus L.J Ecol 1996; 84: 125–132.
21. Uusitalo M. European bird cherry (Prunus padus L.)—a biodiverse wild plant for horticulture. AgrifoodResearcg Reports 61, MTT Agrifood Research Finland, Jokioinen;2004.
22. Vanhellemont M. Present and future population dynamics of Prunus serotina in forests in its introduced range. Ph.D. Thesis, Ghent University. 2009.
23. Halarewicz A.The reasons underlying the invasion of forest communities by black cherry, Prunus serotina and its subsequent consequences. Leś Pr Bad. 2011; 72(3):267–272.
24. Gantner M, Jaśkiewicz B. Beetles (Coleoptera) occurring on hazel (Corylus L.) in different habitat conditions. ActaSci Pol, HortorumCultus. 2002; 1(1): 55–66.
25. Nowakowska K, Halarewicz A. Coleoptera found on neophyte *Prunus serotina* (Ehrh.) within forest community and open habitat. EJPAU. 2006; 9(1): #05.

26. Karolewski P, Giertych MJ, Żmuda M, Jagodziński AM, Oleksyn J. Season and light affect constitutive defenses of understory shrubspecies against folivorous insects.ActaOecol.2013; 53: 19–32.

27. Mardulyn P, Milinkovitch MC, Pasteels JM.Phylogenetic analyses of DNA and allozyme data suggest that *Gonioctena* leaf beetles (Coleoptera; Chrysomelidae) experienced convergent evolution in their history of host-plant family shifts. Syst Biol. 1997; 46(4): 722–747. PMID: 11975339

28. Halarewicz A, Jackowski J.Leaf damage of the black cherry, *Prunusserotina* Ehrh., by the leaf beetle, *Gonioctenaquinquepunctata*Fabr.: an accidental foraging on a neophytic host, or an established trophic link? Pol J Ecol. 2011; 5: 589–597.

29. Leather SR. Insects on bird cherry 1: the bird cherry ermine moth, Yponomeuta evonymellus (L.) (Lepidoptera: Yponomeutidae). EntomolGaz.1986; 37: 209–215.

30. Karolewski P, Jagodziński AM, Giertych MJ, Łukowski A, Baraniak E, Oleksyn J.Invasive Prunus serotina—a new host for Yponomeuta evonymellus (Lepidoptera: Yponomeutidae)? Eur J Entomol. 2014; 111(2): 227–236.

31. Řehounek J. Comparative study of the leaf beetles (Coleoptera: Chrysomelidae)in chosen localities in the district of Nymburk. Acta Univ. Oulu. Ser. A Sci. RerumNatur. 2002; 39–40: 123–130.

32. Bartkowska J. Susówka leszczynowa–*Halticabrevicollis*Foudras, 1860 (Col., Chrysomelidae, Halticinae), potencjalny szkodnik uprawnej leszczyny. Pol J Entomol. 1975; 45: 633–638. (in Polish)

33. Gruev B, Döberl M. General distribution of the flea beetles in Palaearcticsubregion (Coleoptera, Chrysomelidae: Alticinae). Scopolia. 1997; 37: 1–496.

34. Karolewski P, Zadworny M, Mucha J, Napierała-Filipiak A,Oleksyn J.Link between defoliation and root vitality in five understory shrubs with different resistance to insect herbivores. Tree Physiol. 2010; 30: 969–978. doi: 10.1093/treephys/tpq060 PMID: 20624813

35. Paquette A, Bouchard A,Cogliastro A.Morphological plasticity in seedlings of three deciduous species under shelterwood under-planting management does not correspond to shade tolerance ranks. Forest Ecol Manag.2007; 24: 278–287.

36. Giertych MJ, Bąkowski M, Karolewski P, Zytkowiak R, Grzebyta J. Influence of mineral fertilization on food quality of oak leaves and utilization efficiency of food components by the gypsy moth.Entomol Exp Appl. 2005; 117(1): 59–69.

37. Tremmel M, Müller C. Insect personality depends on environmental conditions.Behav Ecol.2013; 24(2): 386–392.

38. Lee KP, Roh C. Temperature-by-nutrient interactions affecting growth rate in an insect ectotherm.Entomol Exp Appl.2010; 136(2): 151–163.

39. Kaplan I, McArt SH, Thaler JS. Plant defenses and predation risk differentially shape patterns of consumption, growth, and digestive efficiency in a guild of leaf-chewing insects. PLOSONE.2014; 9(4): e93714.

40. Pérez-Contreras T, Soler JJ, Soler M. Host selection by the pine processionary moth enhances larval performance: An experiment.ActaOecol.2014; 55: 15–22.

41. Král J. Zur Kenntnis der palaearktischen*Altica*-Arten HI (Col. Phytophaga, Alticidae). IX. Beitrag zur Kenntnis der Alticiden. EntBl Krefeld. 1964; 60: 126–133. (in German)

42. Urban J. A contribution to the knowledge of a chrysomelid beetle *Gonioctena* (= *Phytodecta*) *quinquepunctata* F.(Chrysomelidae, Coleoptera).ActaUniv Agric Silvic Mendel Brun. 1998; 46: 7–23. (in Czech)

43. Blanckenhorn WU.The evolution of body size—what keeps organisms small? Q Rev Biol.2000; 75: 385–407. PMID: 11125698

44. Blanckenhorn WU.Behavioral causes and consequences of sexual size dimorphism. Ethology.2005; 111: 977–1016.

45. Mąderek E, Łukowski A, Giertych MJ, Karolewski P.Influence of native and alien *Prunus* species and light conditions on performance of the leaf beetle *Gonioctena quinquepunctata*. Entomol Exp Appl.2015; 153(3): 193–205.

46. Kudo SI, Ishibashi E, Makino SI.Reproductive and subsocial behaviour in the ovoviviparous leaf beetle *Gonioctenasibirica* (Coleoptera: Chrysomelidae). Ecol Entomol. 1995; 20(4): 367–373.

47. Dixon AFG.The life-cycle and host preferences of the bird cherry-oat aphid, *Rhopalosiphumpadi* L., and their bearing on the theories of host alternation in aphids. Ann Appl Biol. 1971; 68: 135–147. PMID: 5571404

48. Tong YF, Lee SY, Morton B. The herbivore assemblage, herbivory and leaf chemistry of the mangrove *Kandeliaobovata* in two contrasting forests in Hong Kong. WetlEcol Manag.2006; 14: 39–52.

49. Moreau G, Quiring DT, Eveleigh ES,Bauce E.Adventage of mixed diet: feeding on several foliar age classes increases the performance of a specialist insect herbivore. Oceanologia.2003; 135: 391–399.

50. Alonso C, Herrera CM. Seasonal variation in leaf characteristics and food selectionby larval noctuids on an evergreen Mediterranean shrub. ActaOecol.2000; 21(4): 257–265.
51. Jansen M, Stamp NE. Effects of light availability on host plant chemistry and the consequences for behavior and growth of an insect herbivore. Entomol Exp Appl.1997; 82(3): 319–333.
52. Osier T., Jennings S. Variability in host-plant quality for the larvae of a polyphagous insect folivore in midseason: the impact of light on three deciduous sapling species. Entomol Exp Appl. 2007; 123: 159–166.
53. Molina-Montenegro MA, Ávila P, Hurtado R, Valdivia AI, Gianoli E.Leaf trichome density may explain herbivory patterns of *Actinote* sp. (Lepidoptera: Acraeidae) on *Liabummandonii* (Asteraceae) in a montane humid forest (Nor Yungas, Bolivia). ActaOecol.,2006; 30: 147–150.
54. Closset-Kopp D, Chabrerie O, Valentin B, Delachapelle H,Decocq G. When Oskar meets Alice: Does a lack of trade-off in r/K-strategies make *Prunus serotina* a successful invader of European forest? Forest EcolManag. 2007; 247: 120–130.
55. Hemming JD, Lindroth RL.Effects of light and nutrient availability on aspen: growth, phytochemistry, and insect performance. J ChemEcol.1999; 25(7):1687–1714.
56. Mooney EH, Niesenbaum RA.Population-specific responses to light influence herbivory in the understory shrub *Lindera benzoin*. Ecology. 2012; 93(12): 2683–2692. PMID: 23431598
57. Mathur V, Ganta S, Raaijmakers CE, Reddy AS, Vet LEM, van Dam NM. Temporal dynamics of herbivore-induced responses in *Brassica juncea* and their effect on generalist and specialist herbivores. Entomol Exp Appl.2011; 139(3): 215–225.
58. Levesque KR, Fortin M, Mauffette Y. Temperature and food quality effects on growth, consumption and post-ingestive utilization efficiencies of the forest tent caterpillar *Malacosomadisstria* (Lepidoptera: Lasiocampidae). B Entomol Res. 2002; 92(2): 127–136.
59. Wennström A, Hjulström LN, Hjältén J, Julkunen-Tiitto R. Mother really knows best: host choice of adult phytophagous insect females reflects a within-host variation in suitability as larval food. Chemoecology.2010; 20(1): 35–42.
60. Björkman C, Larsson S, Bommarco R.Oviposition preferences in pine sawflies: a trade-off between larval growth and defence against natural enemies. Oikos.1997; 79: 45–52.
61. Heleno RH, Ceia RS, Ramos JA,Memmott J. Effects of alien plants on insect abundance and biomass: a food-web approach. Conserv Biol.2008; 23: 410–419. doi: 10.1111/j.1523-1739.2008.01129.x PMID: 19128322
62. Chase JM, Abrams PA, Grover JP, Diehl S, Chesson P, Holt RD, et al.The interaction between predation and competition: a review and synthesis. Ecol Lett.2002; 5(2): 302–315.
63. Kaplan I,Denno RF. Interspecific interactions in phytophagous insects revisited: a quantitative assessment of competition theory. Ecol Lett.2007; 10(10): 977–994. PMID: 17855811
64. Harvey JA, Fortuna TM. Chemical and structural effects of invasive plants on herbivore–parasitoid/predator interactions in native communities. Entomol Exp Appl. 2012; 144(1): 14–26.
